# Results of a phase II clinical trial of 6-mercaptopurine (6MP) and methotrexate in patients with BRCA-defective tumours

**DOI:** 10.1038/s41416-019-0674-4

**Published:** 2019-12-09

**Authors:** Corran Roberts, Victoria Y. Strauss, Sylwia Kopijasz, Charlie Gourley, Marcia Hall, Ana Montes, Jacinta Abraham, Andrew Clamp, Richard Kennedy, Susana Banerjee, Lisa K. Folkes, Michael Stratford, Shibani Nicum

**Affiliations:** 1 Centre for Statistics in Medicine, Nuffield Department of Orthopaedics, Rheumatology and Musculoskeletal Sciences, University of Oxford, Oxford, UK; 2 Oncology Clinical Trials Office (OCTO), Department of Oncology, University of Oxford, Oxford, UK; 3 Cancer Research UK Edinburgh Centre, MRC IGMM, University of Edinburgh, Edinburgh, UK; 4 Mount Vernon Cancer Centre, Northwood, Middlesex UK; 5 Guy’s and St Thomas’ NHS Foundation Trust, London, UK; 6 Velindre Cancer Centre, Cardiff, UK; 7 The Christie NHS Foundation Trust and Institute of Cancer Sciences, University of Manchester, Manchester, UK; 8 Centre for Cancer Research and Cell Biology, Queen’s University of Belfast, Belfast, UK; 9 The Royal Marsden NHS Foundation Trust and Institute of Cancer Research, London, UK; 10 CRUK/MRC Oxford Institute for Radiation Oncology, Department of Oncology, University of Oxford, Oxford, UK; 11 Oxford University Hospitals NHS Trust, Oxford, UK

**Keywords:** Cancer therapeutic resistance, Cancer therapy

## Abstract

**Background:**

Tumour cells with *BRCA1/2* gene mutations demonstrate increased sensitivity to platinum and poly (ADP-ribose) polymerase (PARP) inhibitors. 6-mercaptopurine (6MP) was found to selectively kill BRCA-defective cells in a xenograft model as effectively as the PARP inhibitor AG014699, even after these cells acquired resistance to a PARP inhibitor or cisplatin.

**Methods:**

This phase II single-arm trial investigated the activity of 6MP 55–75 mg/m^2^ per day, and methotrexate 15–20 mg/m^2^ per week in advanced breast or platinum-resistant ovarian cancer patients with a *BRCA1/2* germline mutation, who had progressed after ≥1 previous line of chemotherapy. The primary outcome was objective response including stable disease (SD) as an assessment of clinical benefit rate (CBR), at 8 weeks, by RECIST v1.1. Secondary outcomes included overall survival (OS) and progression-free survival (PFS).

**Results:**

In total, 67 evaluable patients were recruited; 55 ovarian and 11 breast cancer patients. In total, 21 patients had SD (31%), one had a partial response (1.5%); CBR was 33% at 8 weeks. In total, 12/67 patients (18%) had SD at 16 weeks. In total, five ovarian cancer patients had SD for over 200 days. Median OS was 10.3 months (95% CI 6.9–14.5), median PFS 1.9 months (1.7–2.8).

**Conclusions:**

The overall activity of 6MP and methotrexate in these patients was low; however, there was a small group of patients who appeared to derive longer-term clinical benefit.

**Trial registration:**

NCT01432145 http://www.ClinicalTrials.gov.

## Background

*BRCA1* and *BRCA2* genes play an important role in homologous recombination DNA repair and have been implicated in familial breast and ovarian cancer syndromes. Ovarian cancer is the fifth commonest cancer in women,^1^ with 46% 5-year survival rate.^2^ Over 15% of women who are diagnosed with high-grade serous ovarian carcinoma will have a germline BRCA mutation present.^3,4^ Breast cancer is the most common cancer in women and accounts for between 18 and 25% of all female malignancies worldwide.^5^ There is a familial component in 5–10% of all breast cancer cases, with most commonly, mutations in the *BRCA1/2* genes and *p53*, *ATM* or *PTEN* genes.^6,7^ The triple-receptor negative breast cancer phenotype, i.e. negative for oestrogen receptor, progesterone receptor and HER2, who also carries an adverse prognosis, accounts for 80–90% of BRCA1-associated breast cancers.^8^ For patients with metastatic cancer, the challenge is to develop more effective therapies that maximise tumour cell killing (efficacy) and minimise toxicity.

In patients with BRCA1/2-deficient cancers, the use of molecular targeted therapy by using poly (ADP-ribose) polymerase (PARP) inhibitors, has demonstrated a clear benefit. The molecular mechanisms that underlie the selective killing of homologous recombination-deficient BRCA mutant cells by PARPi were initially thought to be solely due to inhibition of base excision repair (BER), with PARPi causing an increase in DNA single-strand breaks (SSBs) that led to toxic double-strand breaks at replication forks.^9,10^ However, other mechanisms, such as PARP trapping on DNA at sites of unrepaired SSB causing physical obstruction,^11^ and PARPi enhancing non-homologous end joining in some tumour cells,^12^ also may play a significant role in cell death.

PARP inhibitors have revolutionised the treatment of high-grade serous ovarian cancer and have shown particular efficacy in women with a BRCA mutation. Between 2014 and 2017, three PARP inhibitors, olaparib (LYNPARZA®, AstraZeneca Pharmaceuticals LP^13^), niraparib^14^ and rucaparib^15^ have been licensed in the treatment of recurrent high-grade ovarian cancer. Olaparib has recently shown efficacy in the front-line setting, with an improvement in disease-free survival when used as a maintenance therapy trial in women with newly diagnosed ovarian cancer, which may result in a new treatment option in the near future.^16^

Among patients with HER2-negative metastatic breast cancer and a germline BRCA mutation, olaparib monotherapy provided a significant benefit over standard therapy; median progression-free survival was 2.8 months longer and the risk of disease progression or death was 42% lower with olaparib monotherapy than with standard therapy.^17^

There are multiple mechanisms of PARP inhibitor resistance, including restoration of the homologous recombination pathway through secondary BRCA reversion mutations,^18^ hyperactivation of non-homologous end joining^19^ and increased stabilisation of replication forks independent of BRCA1/2 reversion mutations.^20^ Given the expanding clinical use of PARP inhibitors and the high likelihood of acquired resistance, there is a significant need for new treatment strategies to manage PARP inhibitor-resistant disease.

In a screen for novel drugs that selectively kill BRCA2-defective cells, Helleday and colleagues identified 6-thioguanine (6TG)^21^ and demonstrated that 6TG induces DNA double-strand breaks that are repaired by homologous recombination. They found that 6TG was as efficient as the PARP inhibitor, AG014699, in selectively killing BRCA2-defective tumours in a xenograft model, and that 6TG also kills cisplatin-resistant or PARP inhibitor-resistant (PIR) BRCA2-defective cells.^21^ Although homologous recombination is reactivated in some PIR cells in response to PARP inhibitors, it is not fully restored for the repair of 6TG-induced lesions. This is likely to be due to the repair of 6TG defects also being dependent on mismatch repair (MMR), in contrast to the MMR-independent replication defects produced by PARP inhibitors. This suggested that 6TG may be effective in the treatment of tumours that have developed resistance to PARP inhibitors or cisplatin chemotherapy.^21^

6-Mercaptopurine (6MP) is a prodrug that is converted to the same cytotoxic moiety as 6TG, i.e. 6-thioguanine nucleotides (6TGN), but with fewer toxic effects.^22^ Low-dose methotrexate is used in combination with 6MP as it promotes the formation of 6TGN. 6MP is metabolised to 6-thioinosine monophosphate and subsequently to 6TGN by hypoxanthine guanine phosphoribosyl transferase and can then be incorporated into nucleic acids. See Supplementary Fig. 1. The cytotoxic effects of 6MP are predominantly due to this incorporation of 6TGN into the DNA, as they are structurally similar to endogenous purine-based guanine. Thiopurine toxicity is delayed as TGNs require passage through one S phase of the cell cycle to incorporate into DNA in place of guanine. The incorporated 6TGN (about 1 in 104 bases) is methylated to 6-meTG by endogenous S-adenosylmethionine, which becomes a substrate for mismatch repair in the second replication round to mediate its toxicity.^23–26^

Inactivation of 6MP to 6-thiouric acid occurs via xanthine oxidase and also by thiopurine methyltransferase (TPMT). TPMT methylates 6MP to 6-methylmercaptopurine (6MMP) at the expense of TGNs and 6MMP is a strong inhibitor of purine de novo synthesis and can also result in immunosuppression.^27^ Quantitation of 6TG (activation of 6MP) and 6MMP (inactivation of 6MP) may give an indication of TPMT activity and hence the potential efficacy of 6MP in a patient.^28^

It can take 2–3 months (depending on TPMT status) to see therapeutic benefits from 6MP in acute lymphoblastic leukaemia (ALL) and inflammatory bowel disease.^29^

Methotrexate is also an antimetabolite antineoplastic agent. It inhibits tetrahydrofolate dehydrogenase and prevents formation of tetrahydrofolate, which is required for synthesis of thymidylate, an essential component of DNA. It is routinely used in combination with 6MP in patients with ALL as it has been shown to reduce de novo purine synthesis and thus enhance the cytotoxicity of 6MP by promoting its conversion to 6TGNs.^30^ Methotrexate has also been widely used in a number of solid tumours such as breast, ovarian, lung and cervical cancers and shown to induce regression. Furthermore, a synthetic lethality screen has identified methotrexate as an agent that has activity in DNA MMR-defective cancers, as evidenced by its selective toxicity in cells lacking functional mutS homologue 2 (MSH2) gene mutations.^31^

Based on these preclinical findings, 6MP, combined with low-dose methotrexate, was used to test a new therapeutic option for women with known *BRCA* mutations and relapsed breast or ovarian cancer. This is the first trial to assess the efficacy and toxicity of 6MP and weekly methotrexate in women with *BRCA*-mutated platinum-resistant ovarian cancer or relapsed *BRCA*-mutated breast cancer.

## Methods

### Study design

The 6MP study was a single-arm, two-stage Phase II trial, which recruited from 14 centres across the United Kingdom. Conduct of the trial complied with the Declaration of Helsinki, and ethical approval was obtained prior to the study opening.

Further details of the study design, patient selection, interventions and outcome measures are provided in the previously published protocol paper.^32^

### Participants and treatment

To be eligible for inclusion in the 6MP study, patients must have been aged 18 or older, have proven *BRCA1* or *BRCA2* germline mutations and measurable disease as defined by Response Evaluation Criteria in Solid Tumours (RECIST) v1.1 criteria. Ovarian cancer patients had to have disease that was either platinum-resistant or be those in whom further platinum-based therapy was inappropriate. Breast cancer patients must have had locally advanced or metastatic breast cancer and may have received up to three previous lines of chemotherapy in the locally advanced or metastatic setting. All patients must have had an Eastern Cooperative Oncology Group (ECOG) performance score between 0 and 2, a life expectancy of greater than 12 weeks and adequate haematological and biochemical function. Patients were excluded from participating in the study if they had a Low/Low genotype on TPMT testing.

Registered patients received 6MP once daily and methotrexate once weekly in 28-day cycles until they developed disease progression, unacceptable toxicity or patient/clinician decision to stop treatment. Starting doses were modified from 75 mg/m^2^ of 6MP and 20 mg/m^2^ of methotrexate to 55 and 15 mg/m^2^, respectively, because many of the first 26 registered patients required dose reductions or treatment delays due to myelosuppression.

### Outcome measures

Primary outcome was objective response including stable disease (SD), as an assessment of clinical benefit (CB), according to RECIST v1.1, defined as complete response (CR), partial response (PR) and stable disease confirmed by computerised tomography or magnetic resonance imaging. Tumour assessments were carried out at baseline and every 8 weeks until the end of treatment. Quality assurance of tumour assessments was conducted by an independent radiographer for at least one patient at each site. Two time points of interest were 8 and 16 weeks after the first treatment. Long-term stable disease patients were defined as those having SD for over 200 days. Secondary endpoints included overall survival (OS), progression-free survival (PFS), pharmacokinetics (PK) and quality of life (QoL). Outcome definitions are provided in the protocol paper. Safety was evaluated by using National Cancer Institute Common Terminology Criteria for Adverse Events (CTCAE) version 4.0. Feasibility of a multicentre trial was evaluated by at least three patients per year per site. A CA125 response according to the Gynaecological Cancer Intergroup (GCIG) criteria occurred if there was at least a 50% reduction in CA125 levels from a pre-treatment sample and this was maintained for at least 28 days.

### Sample size determination

The Simon compromise/admissible two-stage design^33,34^ was implemented to provide enough power for an estimated 65 evaluable patients to detect a 10% change from 10% in the absolute response rate including SD with a power of 90% and a significance level of 20%.

### Statistical analysis and patient groups

The proportions of clinical benefit and of CR, PR and SD at 8 and 16 weeks were described. Association of objective response at 8 weeks with baseline characteristics (prior PARP inhibitor treatment, *BRCA* status, TPMT status and volume of disease) was assessed by using a chi-squared test or Fisher’s exact test, as appropriate.

OS and PFS were presented as median and quartiles overall and separately for patients previously treated with PARP inhibitors. A Tarone–Ware test rather than a log-rank test was applied to assess the association of OS and PFS with prior PARP inhibitor treatment because of the indication of non-proportional hazards.

Objective response rate (ORR), stable disease rate, OS and PFS analyses were conducted on a modified intention-to-treat population, which included evaluable patients who had received at least one dose of treatment and who had a response assessment, irrespective of their compliance to the planned course of treatment.

The safety analysis population comprised all registered patients.

6MMP (inactivation of 6MP) and 6TG (activation of 6MP) were measured in red blood cells for pharmacokinetic analysis, by using a validated high-performance liquid chromatography method (HPLC) with absorbance detection using similar methods as described.^35^ Red blood cell (RBC) 6TG and 6MMP was assessed at Cycle 1 days 1 and 8, Cycle 2 day 1 and Cycle 3 day 1. Acid hydrolysis is used to convert 6MMP and 6TGN to AMTCI and 6TG, respectively, this is detectable at wavelengths of 310 and 342 nm, respectively. The ratio of 6TG and 6MMP could give an indication of TPMT activity and hence the efficacy of 6MP in a patient. The inter-patient variability in 6MP and 6TG levels was assessed graphically as well as their relationship with response over time. TPMT status was assessed in all patients.

## Results

### Patient demographics and recruitment feasibility

**Fig. 1:**
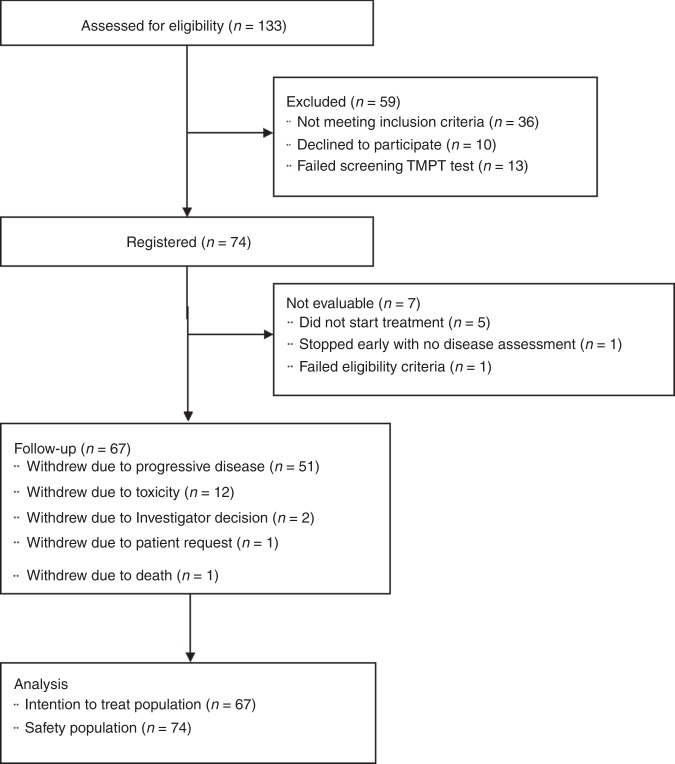
CONSORT flow diagram.

In total, 133 patients with advanced ovarian or breast cancer were screened for eligibility from 14 UK sites between May 2011 and October 2014; 74 patients were consented and registered, and 67 of these registered patients were found to be evaluable (Fig. 1). This is larger than the planned sample size of 65 patients, to compensate for unevaluable patients. On average, 1.4 patients were recruited per site per year.

Baseline characteristics are shown in Table 1. Of the 67 evaluable, 57 (85%) were ovarian cancer patients and 10 (15%) had breast cancer. In total, 40 (60%) patients had a *BRCA1* mutation and 27 (40%) had a *BRCA2* mutation. Of all the patients, 26 (39%) had received a prior PARP inhibitor. These patients were heavily pre-treated, and the mean number of prior therapies received for ovarian cancer was 4.7 (standard deviation (STD) 2.2), and 4.2 (STD 2.3) for breast cancer. The median time between finishing previous therapy and entering the 6MP trial was just 1.9 (interquartile range (IQR) 1.1–4.6) months across all patients.

**Table 1:** Baseline characteristics.

Target cancer site	All	Ovarian cancer	Breast cancer
	*N* = 67	*N* = 57	*N* = 10
	*n* (%)	*n* (%)	*n* (%)
Age (years) mean (range, SD)	55.9 (32–80, 10.5)	57.7 (35–80, 10.0)	46.1 (32–59, 8.3)
Gender			
Male	0	0	0
Female	67 (100%)	57 (100%)	10 (100%)
Mutated *BRCA* gene			
1	40 (60%)	36 (63%)	4 (40%)
2	27 (40%)	21 (37%)	6 (60%)
Platinum-resistant disease			
Yes	49 (73%)	49 (86%)	0
No	8 (12%)	8 (14%)	0
N/A (breast cancer patient)	10 (15%)	0	10 (100%)
Prior PARP treatment			
Yes	26 (39%)	24 (42%)	2 (20%)
No	41 (61%)	33 (58%)	8 (80%)
No. of prior therapies mean (range, SD)		4.7 (1–8, 2.2)	4.2 (1–8, 2.3)
ECOG performance status			
0	27 (40%)	22 (39%)	5 (50%)
1	36 (54%)	31 (54%)	5 (50%)
2	4 (6%)	4 (7%)	0
Albumin levels, mean (range, SD)	39.8 (28–49, 5.4)	39.1 (28–49, 5.4)	43.7 (39–49, 3.6)
<35 g/dl	15	15	0
≥35 g/dl	52	42	10
Volume of disease			
Visceral*	34 (51%)	26 (46%)	8 (80%)
Bulky AP > 2 cm**	24 (36%)	24 (42%)	0
AP < 2 cm ± nodal	4 (6%)	4 (7%)	0
Nodal only	5 (7%)	3 (5%)	2 (20%)
TPMT mean (range, SD)	88.3 (43–160, 19.2)	85.8 (43–135, 17.7)	102.2 (84–160, 22.6)
<68 mU/L	6	6	0
≥68 mU/L	61	51	10

*Included patients both with and without bulky AP > 2 cm, AP < 2 cm and nodal disease.

**Included patients both with and without AP < 2 cm and nodal disease.

### Treatment exposure

Median total duration of trial treatment was 55 days overall; 55 days for ovarian cancer patients and 91 days for breast cancer patients. In total, 18 patients (27%) remained on the trial for over 100 days, and 7 patients (10%) for over 200 days. In total, 5 patients (7%) discontinued treatment within 4 weeks due to disease progression. Among the 24 patients who started with 75 mg/m^2^ of 6MP and 20 mg/m^2^ of methotrexate, 11 (46%) required dose modifications. Their mean daily 6MP dose intensity was 94.4 mg/day (STD 29.9) and their median time on trial was 63 days (IQR 30–135). After modification of the starting dose, 10 of the remaining 43 patients (23%) required dose modifications, their mean daily 6MP dose intensity was 82.6 mg/day (STD 23.6) and their median time on trial was 55 days (IQR 40–61).

### Objective response (clinical benefit)

At stage 1 of the trial, the first milestone of at least 3 out of 30 evaluable patients having SD, PR or CR at 8 weeks was surpassed; 10/30 patients (33%) had SD at 8 weeks, and hence the trial continued to recruit a further 35 patients.

Of 67 evaluable patients at the second stage, 22 (CBR: 33% with 95% CI: 22–45%) patients had an objective response or stable disease at 8 weeks. Only one patient had a partial response; hence the stable disease rate was 31% (Table 2). At 16 weeks, 12 (18%) patients had SD. No statistically significant correlation between 8- or 16-week response/disease stabilisation and any baseline characteristics, such as *BRCA* status, ECOG performance status or volume of disease, was found.

**Table 2:** Tumour RECIST response rates at 8 and 16 weeks.

	Target cancer site	All		Ovarian cancer		Breast cancer	
Timepoint	Response	*N* = 67		*N* = 57		*N* = 10	
		No.	%	No.	%	No.	%
8 weeks	CBR (CR, PR and SD)	22	33	17	30	5	50
	CR	0	0	0	0	0	0
	PR	1	1	0	0	1	10
	SD	21	31	17	30	4	40
	PD	22	33	18	32	4	40
	Withdrew prior to scan	23	34	22	39	1	10
16 weeks	Objective response (CR, PR and SD)	12	18	8	14	4	40
	CR	0	0	0	0	0	0
	PR	0	0	0	0	0	0
	SD	12	18	8	14	4	40
	PD	6	9	5	9	1	10
	Withdrew prior to scan	49	73	44	77	5	50

*CBR* clinical benefit rate, *CR* complete response, *PR* partial response, *SD* stable disease, *PD* progressive disease

### Biochemical response rate

In total, 6/57 (11%) ovarian cancer patients had a CA125 response by GCIG criteria over the course of the trial.

### Progression-free and overall survival

**Fig. 2:**
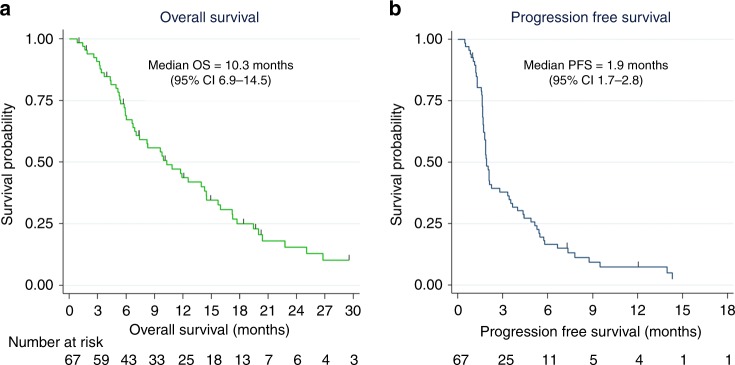
Overall survival (**a**) and progression-free survival (**b**).

Median OS was 10.3 months (95% CI 6.9–14.5 months). Median PFS was 1.9 months (95% CI 1.7–2.8) and the proportion of patients who were progression free at 6 months was 16%. OS and PFS Kaplan–Meier curves are shown in Fig. 2a, b, respectively.

### Response by prior PARPi exposure

There were no statistically significant differences in OS or PFS in those 26 patients (39%) who had received prior PARP inhibitor treatment compared with those 41 patients (61%) who had not (Tarone–Ware test, *p* = 0.84 for OS, *p* = 0.93 for PFS). The median OS for patients exposed and unexposed to prior PARP were 9.9 (5.9–17.7 months) and 11.9 months (7.1–14.5 months). The median PFS values were 2.1 (1.7–3.4) and 1.8 (1.6–3.7).

### Longer-term stable disease patients

In total, 5 ovarian cancer patients had SD for more than 200 days; their clinical characteristics are summarised in Table 3. All but one of these patients had visceral disease. Two patients also had a biochemical response according to GCIG criteria. One patient had just one Grade 3 or 4 AE during the course of the trial, and the other four patients had between three and five Grade 3 or 4 AEs.

**Table 3:** Clinical characteristics of the five ovarian cancer patients with stable disease for over 200 days.

	Baseline characteristics				6MP study treatment details		
Patient ID	Mutated *BRCA* gene	Prior PARP treatment	Volume of disease	TPMT status	6MP starting dose (mg)	Mean daily 6MP dose intensity (mg)	No. of days on trial
Patient 1	1	Yes	Visceral	High/high	140	98.1	303
Patient 2	1	No	Visceral	High/high	130	93.2	287
Patient 3	2	Yes	Visceral	High/high	120	58.7	260
Patient 4	1	Yes	Nodal only	High/high	110	98.3	455
Patient 5	2	No	Visceral	High/high	100	67.6	252*

*Still on treatment at the time of analysis.

### Tolerability

A pre-planned safety analysis was performed after the first 12 patients had received 3 months of therapy. The initial starting dose was 75 mg/m^2^ of 6MP and 20 mg/m^2^ of methotrexate. In total, 11 of the first 24 patients (46%) required a dose reduction, and therefore the starting dose was reduced to 55 mg/m^2^ of 6MP and 15 mg/m^2^ of methotrexate. Subsequently 11 of 43 patients (26%) required a dose reduction.

In total, 67 (91%) of 74 registered patients had at least one AE, of which 45 patients (61%) had 106 AEs of CTCAE grade 3 or above. The most frequently occurring grade 3 or above AEs were abdominal pain, anaemia and neutropenia. In total, 23 episodes of grade 3–4 neutropenia were reported in the trial, experienced by 18/74 (24%) patients (Table 4). There were also 167 CTCAE grade 2 adverse drug reactions, experienced by 48/74 (65%) patients, most commonly anaemia, fatigue, neutropenia and nausea. SAEs were reported by 33 (49%) patients; 47 SAEs in total, of which 12 were Grade 3 or above. There were no treatment-related deaths; all deaths were disease related.

**Table 4:** Grade 2 adverse drug reactions and Grade 3–4 adverse events reported in ≥10% of patients overall.

Event term	Grade 2 adverse drug reactions*	Grade 3–4 adverse events	Total
Abdominal pain	3	6	9
Alanine aminotransferase increased	5	4	9
Anaemia	24	6	30
Fatigue	22	5	27
Mucositis oral	7	0	7
Nausea	15	4	19
Neutrophil count decreased	19	23	42
Platelet count decreased	4	4	8
Vomiting	7	5	12
White blood cell decreased	13	8	21
Total	119	65	184

*An adverse drug reaction is an AE that is considered to be causally related to any dose of the 6MP or methotrexate.

Over the course of the trial, 12 patients (18%) were withdrawn from study treatment due to toxicity. Other causes of treatment discontinuation, without PFS, were investigator decision (2 patients, 3%), patient request (1 patient, 2%) and death (1 patient, 2%). All other patients (76%) had a PFS event.

### Pharmacokinetics

#### Measurement of 6TG and 6MMP

Our analysis demonstrated considerable inter-patient variability in the levels of RBC 6TG and 6MMP levels (Supplementary Fig. 2). There was no apparent relationship between the levels of the active moiety (6TGN measured as 6TG) and response to 6MP or duration of response or PFS. The wide inter-patient variability in 6TGN levels observed is thought likely to be as a result of genetic polymorphisms of TPMT.

#### Pharmacogenomics (TPMT status)

The TPMT status in the subset of patients who derived the greatest clinical benefit, extending beyond 200 days, demonstrated that all of the five long-term stable disease patients had high/high TPMT status.

### Quality of life (QoL)

Quality of life could not be analysed due to the low questionnaire completion rate; only two patients (3%) completed the baseline and 12-month follow-up QoL questionnaires.

## Discussion

The aim of this trial was to determine the activity and efficacy of 6MP in combination with weekly methotrexate in women with relapsed BRCA-mutated breast and ovarian cancer.

Despite the fact that 6MP 55 mg/kg and MTX 15 mg/m^2^ was a tolerable combination, there was only one partial response to treatment. However, disease stabilisation (8 weeks) was seen in 33% (95% CI 22–45%) of patients and a small proportion of patients (*n* = 5, 7%) derived disease stabilisation and clinical benefit (>200 days) with this combination. The response rates and stable disease rates observed are lower than the rates observed in a single-arm phase II trial of the PARP inhibitor, olaparib, that demonstrated a 31% response rate by RECIST v1.1 and a 40% disease stabilisation rate in women with *BRCA*-mutated platinum-resistant ovarian cancer, and a 12.9% (95% CI, 5.7–23.9) response rate and 47% (95% CI, 34.0–59.9) stable disease rate in advanced breast cancer.^36^

In comparison with standard chemotherapy in this setting, i.e. women with advanced platinum-resistant ovarian cancer, the SaPPrOC trial demonstrated a 43% response rate (combined CA125 and RECIST response) and PFS of 5.3 months in women treated with weekly paclitaxel.^37^ Retrospective data indicate similar response rates to weekly paclitaxel in women with *BRCA-* mutated ovarian cancer.^38^ Furthermore, a trial of caelyx chemotherapy vs olaparib (200 vs 400 mg) in women with BRCA- mutated ovarian cancer that had recurred within 12 months of prior platinum chemotherapy demonstrated a PFS of 7.1 months in the group receiving caelyx.^39^

Despite the use of cross-trial comparisons, it is clear that the clinical efficacy of 6MP with MTX is lower in comparison with standard chemotherapeutic agents.

### TPMT status and TGN levels

We hypothesised that higher levels of intracellular 6TGN may result in increased efficacy of 6MP due to the long half-life of these metabolites.^40^ Our study demonstrated significant inter-patient and intra-patient variability in the levels of RBC 6TGN (measured as 6TG). The main determinants of 6MP activity have been found to be BSA and TPMT mutational status.^41,42^ We assessed TPMT status and excluded those patients homozygous for TPMT, due to the high chance of life-threatening myelosuppression.^43^ In this study, we did not demonstrate a correlation between dose intensity and TPMT status. We also did not alter 6MP dose based on TPMT status, white blood cell (WBC) counts or RBC TGN levels as is recommended in the treatment of paediatric ALL.^44^ In fact, following the pre-planned safety review in the first 12 patients, a dose reduction from 75 to 55 mg/m^2^ was required due to the high rates of myelosuppression and dose reductions required. It is likely that this cohort of heavily pre-treated patients, with an average of 4.7 (ovarian) and 2.8 (breast) prior lines of therapy, had significantly reduced bone marrow capacity compared with paediatric ALL patients.

The five long-term SD ( > 200 days) all had high/high TPMT status and experienced similar toxicity to non-long-term responders. TPMT is the predominant enzyme involved in methylation and formation of inactive metabolites of 6MP, and it could therefore be expected that these patients may have lower levels of RBC TGN and higher levels of 6MMP and therefore reduced activity of 6MP. This is contrary to the results seen in paediatric ALL patients, where the greatest efficacy is seen in the heterozygote TPMT group and where dose escalation of 6MP has been shown to be of benefit.^27^ However, the main methylation product, 6MMP, has been shown to be a strong inhibitor of purine de novo synthesis and also causes immunosuppression,^27^ and therefore this group (high/high TPMT status) may have thus had greater anticancer effects from treatment. Studies in ALL patients have demonstrated that the greater the inhibition of de novo purine synthesis, the greater the anti-leukaemic effects observed.^45^ We might also have expected decreased toxicity in this subgroup of patients, due to the increased formation of inactive metabolites due to their high TPMT status.

It can take 2–3 months (depending on TPMT status) to see therapeutic benefits from 6MP in ALL and inflammatory bowel disease.^29^ In our study, five patients (7%) discontinued treatment within 4 weeks and 27 patients (40%) discontinued within 8 weeks due to disease progression, suggesting that there was inadequate initial disease control with 6MP with MTX. This is in comparison with olaparib, where therapeutic effects are seen within a few weeks. It may therefore have been better to consider the use of this combination as a maintenance therapy post chemotherapy, rather than upfront treatment, due to the increased time 6MP takes to reach therapeutic levels compared with agents such as olaparib or chemotherapy.

### PARPi resistance

Preclinical data demonstrated activity of 6MP in PARP inhibitor-resistant cells and we therefore included patients who had previously received PARP inhibitors. Planned subgroup analysis of the 26 patients (39%) who had received prior PARP inhibitor therapy was performed and compared with the 41 patients (61%) who were PARP inhibitor naive. No difference in efficacy was demonstrated in the two patient groups to support the preclinical findings. However, this might be due to the lack of statistical power in the subgroup analyses.

### Study limitations

This was a group with heavily pre-treated patients, who had 4.7 (ovarian) and 4.2 (breast) prior lines of therapy, and therefore there was a limited chance of seeing benefit in this group. Furthermore, the time taken for 6MP to reach therapeutic levels may also have contributed to the low response rates seen at 8 weeks.

We used 6TGN incorporation into RBCs as a surrogate for tumour and WBC levels of incorporation of active cytotoxic metabolites. Whilst there is a good correlation between RBC TGN levels and lymphocyte TGN levels, there is less evidence with respect to tumour levels of active metabolites. As patients did not have biopsies whilst on trial, we were unable to determine intra-tumoral levels of active TGNs.

### Final conclusions

This was the first trial to investigate the use of a novel, cost-effective combination of 6MP and MTX in patients with multiple relapsed breast and ovarian cancer with a known BRCA mutation. Although overall the activity of this combination was low, there was a small subset of patients who derived longer-term clinical benefit. There may have been enhanced activity if we had used 6MP/MTX as maintenance therapy or individually dose escalated based on TPMT status, as in the paediatric ALL practice. However, dose escalation is more problematic in patients who have had multiple lines of prior treatment compared with the paediatric ALL setting, where this approach is standard, and we therefore chose a flat dosing schedule.

There are now a number of ongoing trials in this setting with PARP inhibitors both as a single agent and in combination with agents such as cediranib (e.g. the OCTOVA trial), and immune checkpoint inhibitors. Epithelial ovarian cancers that develop in patients with BRCA (germline and somatic) mutations have been shown to have a higher mutational load, leading to an increase in the number of neoantigens and to higher levels in tumour-infiltrating lymphocytes, CD3+ and CD8+ counts and higher levels of PD-1 and PD-L1.^46,47^ This group of patients may therefore derive increased benefit from immune checkpoint inhibitors, either as single-agent therapy or in combination with PARP inhibitors or chemotherapy. Similarly, combination approaches of cisplatin-based chemotherapy and immunotherapy may also be beneficial in women with BRCA-mutated triple-negative breast cancer.^48^ In view of these developments, it is unlikely that further investigation of this combination will be feasible.

## Supplementary information

Supplementary Information

## Data Availability

The datasets used and/or analysed during this study are available from the corresponding author on reasonable request with permission of 6MP trial management group committee.
