# Supplementary material for: Results of a phase II clinical trial of 6-mercaptopurine (6MP) and methotrexate in patients with BRCA-defective tumours
Source: Br J Cancer. 2019 Dec 9;122(4):483–90. doi: 10.1038/s41416-019-0674-4 (PMC7028724; doi:10.1038/s41416-019-0674-4)
Supplement: Supplementary file 1 — Supplementary Information [file 41416_2019_674_MOESM1_ESM.docx]

# Supplementary Information

Figure 1: Thiopurine metabolism


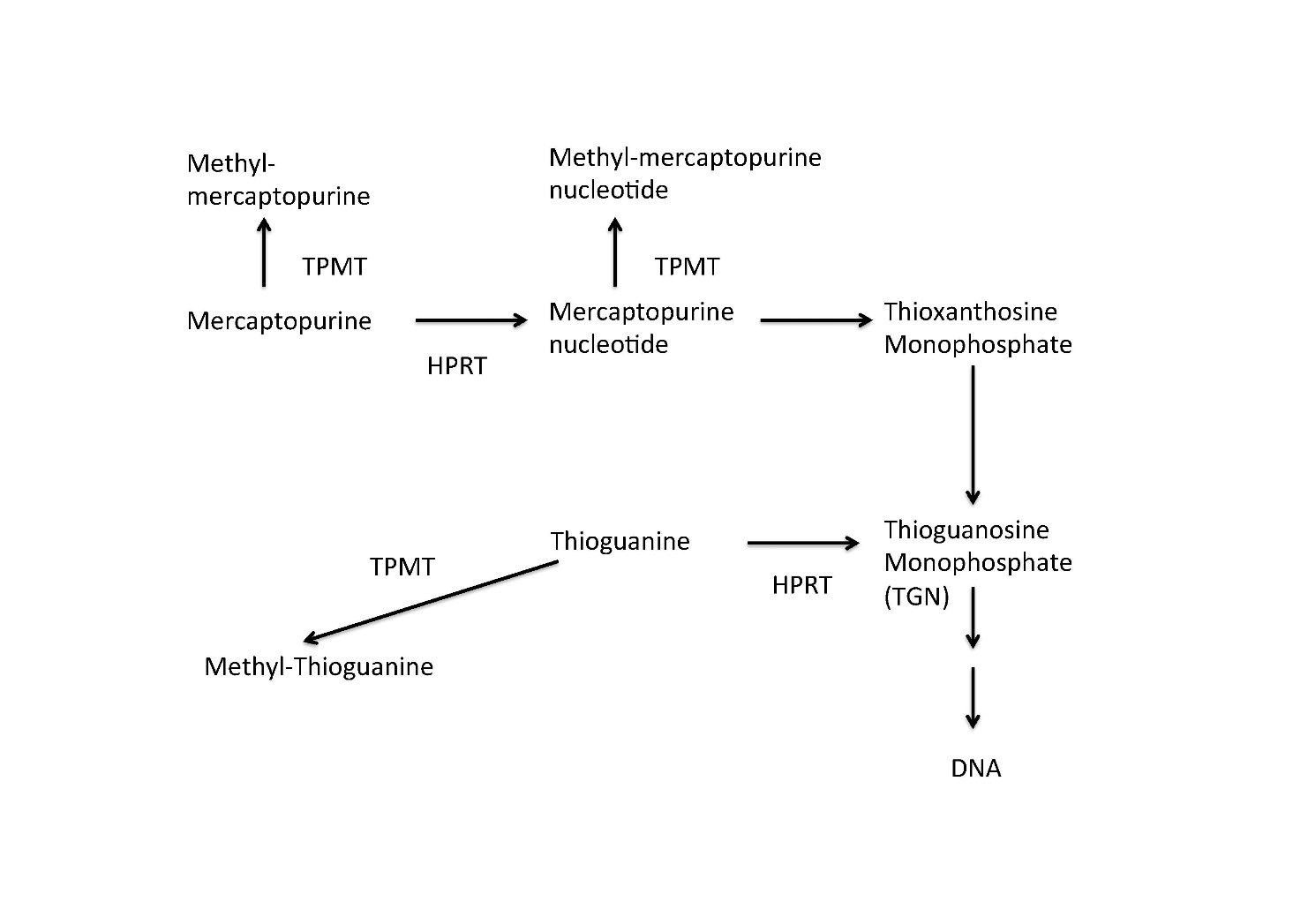


Figure 2: Concentration of 6MMP and 6TG in Red Blood Cells (RBCs)
